# Accuracy of real-time respiratory motion tracking and time delay of gating radiotherapy based on optical surface imaging technique

**DOI:** 10.1186/s13014-020-01611-6

**Published:** 2020-07-10

**Authors:** Li Chen, Sen Bai, Guangjun Li, Zhibin Li, Qing Xiao, Long Bai, Changhu Li, Lixun Xian, Zhenyao Hu, Guyu Dai, Guangyu Wang

**Affiliations:** 1 Department of Radiation Oncology, Cancer Center and State Key Laboratory of Biotherapy, West China Hospital, Sichuan University, Chengdu, China; 2 School of Physics and Technology, Wuhan University, Wuhan, China

**Keywords:** Surface-guided radiation therapy, Optical surface imaging, Respiratory motion tracking, Gating radiotherapy, Time delay

## Abstract

**Background:**

Surface-guided radiation therapy (SGRT) employs a non-invasive real-time optical surface imaging (OSI) technique for patient surface motion monitoring during radiotherapy. The main purpose of this study is to verify the real-time tracking accuracy of SGRT for respiratory motion and provide a fitting method to detect the time delay of gating.

**Methods:**

A respiratory motion phantom was utilized to simulate respiratory motion using 17 cosine breathing pattern curves with various periods and amplitudes. The motion tracking of the phantom was performed by the Catalyst™ system. The tracking accuracy of the system (with period and amplitude variations) was evaluated by analyzing the adjusted coefficient of determination (A_R^2^) and root mean square error (RMSE). Furthermore, 13 actual respiratory curves, which were categorized into regular and irregular patterns, were selected and then simulated by the phantom. The Fourier transform was applied to the respiratory curves, and tracking accuracy was compared through the quantitative analyses of curve similarity using the Pearson correlation coefficient (PCC). In addition, the time delay of amplitude-based respiratory-gating radiotherapy based on the OSI system with various beam hold times was tested using film dosimetry for the Elekta Versa-HD and Varian Edge linacs. A dose convolution-fitting method was provided to accurately measure the beam-on and beam-off time delays.

**Results:**

A_R^2^ and RMSE for the cosine curves were 0.9990–0.9996 and 0.110–0.241 mm for periods ranging from 1 s to 10 s and 0.9990–0.9994 and 0.059–0.175 mm for amplitudes ranging from 3 mm to 15 mm. The PCC for the actual respiratory curves ranged from 0.9955 to 0.9994, which was not significantly affected by breathing patterns. For gating radiotherapy, the average beam-on and beam-off time delays were 1664 ± 72 and 25 ± 30 ms for Versa-HD and 303 ± 45 and 34 ± 25 ms for Edge, respectively. The time delay was relatively stable as the beam hold time increased.

**Conclusions:**

The OSI technique provides high accuracy for respiratory motion tracking. The proposed dose convolution-fitting method can accurately measure the time delay of respiratory-gating radiotherapy. When the OSI technique is used for respiratory-gating radiotherapy, the time delay for the beam-on is considerably longer than the beam-off.

## Introduction

Respiratory motion is a major source of target uncertainty in the external radiation treatment of thoracic and abdominal tumors [1–3]. This results in deviation in dose distributions. Hence, a target may not receive adequate dose coverage while a normal tissue may be exposed to higher doses as planned [4–6]. The uncertainty caused by respiration could be minimized by respiratory motion management [7, 8]. At present, the commonly used respiratory management methods in radiation oncology include motion-encompassing methods, respiratory-gating methods, breath-hold methods, forced shallow-breathing with abdominal compression, and respiration-synchronized methods [9]. All such methods require precise tumor localization and tracking using imaging techniques, particularly respiratory-gating methods and respiration-synchronized methods.

Surface-guided radiation therapy (SGRT) is a non-invasive imaging guided technique that can provide real-time motion tracking of the chest/breast surface which can be used as a surrogate for internal tumors [10], without additional radiation [11]. It could also be used for generating a continuous respiratory signal for gating treatment. Currently, several OSI systems are available for clinical use, such as Align RT (Vision RT, London, United Kingdom), Catalyst™ (C-Rad, Upsalla, Sweden), and Identify (Varian Medical System, Inc., U.S).

SGRT has been applied in clinics [12–14]. At present, the SGRT technique is commonly used to assist with patient set-up but is beginning to be used in motion tracking under static states, such as positioning breast cancer radiotherapy with the deep inspiration breath-hold (DIBH) technique, and stereotactic radiosurgery [15–17]. However, there are limited studies on the precision of respiratory motion tracking, which is crucial for respiratory-gating and respiration-synchronized methods.

The main commercially available systems for non-radiographic localization and the tracking systems for body surfaces are infrared systems and optical systems. The research on respiratory motion tracking [18–20] focuses primarily on infrared systems. The results suggest that fitted and clinical respiratory curves provide high real-time motion tracking accuracy and the Pearson correlation coefficient (PCC) is greater than 0.9. However, when using an infrared system, an infrared reflector must be placed on a device or on a patient. Additionally, a direct line of sight must be maintained between the reflector and camera. If an obstacle exists between the camera and the reflector, the system will obtain inaccurate position information. However, an optical system can effectively overcome these shortcomings. The Catalyst™ system directly tracks the signals of the three-dimensional images of the body surface using three probes. In this work, we have explored the influences of period and amplitude variations and respiratory patterns on the accuracy of real-time motion tracking using the OSI technique.

Respiratory gating can potentially reduce the planning target volume margin and therefore reduce normal tissue toxicity [20–22]. However, this method has inherent inaccuracies [23–25] . One of these inaccuracies is the time delay, which may lead to treatment inefficiencies and “geographic miss” [26]. In this regard, the time delay is the most basic parameter that controls delivery accuracy. According to AAPM TG report 142, when the tumor movement speed is 2 cm/s, the corresponding time delay should not exceed 100 ms [27]. Therefore, the time delay should be measured before using the SGRT technique in respiratory gating radiotherapy. Even though the time delay of respiratory gating has been investigated using the single-exposure method [20, 25, 26], there are certain limitations in the measurement of the time delay. In this work, we have proposed an accurate mathematical method to measure the time delay of the Versa-HD (Elekta Instrument AB Stockholm, Sweden) and Edge (Varian Medical System, Inc., U.S) linacs in respiratory-gating radiotherapy based on the SGRT technique.

## Materials and methods

### Optical surface imaging system and QUASAR programmable respiratory motion phantom

**Fig. 1:**
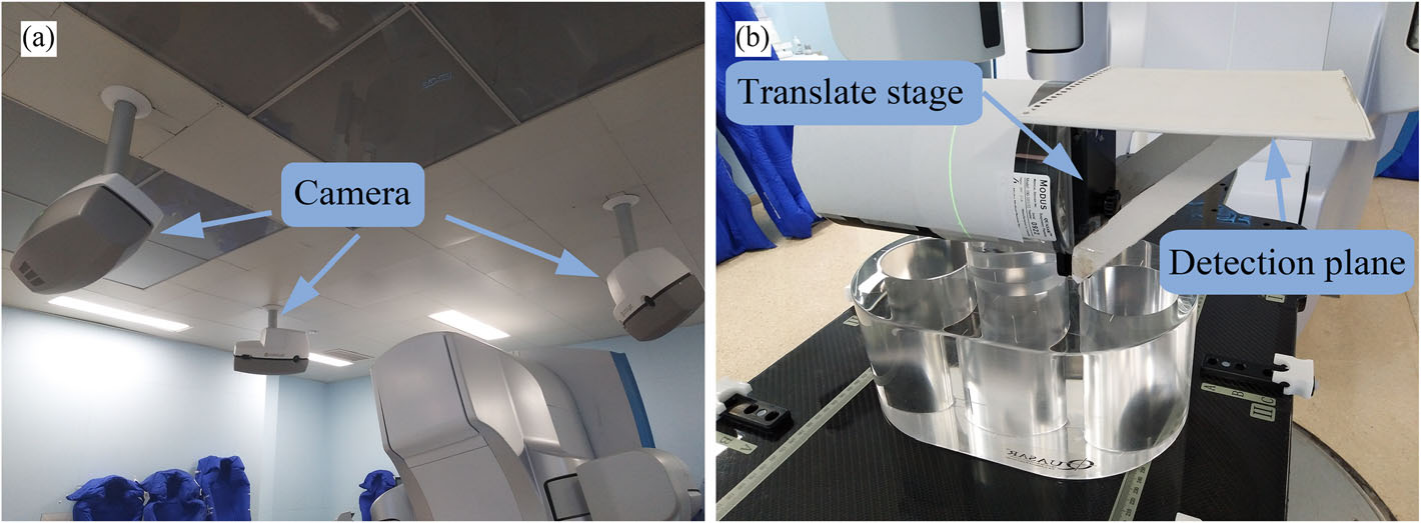
**a** Catalyst™ optical surface imaging system and **b** modified QUASAR programmable respiratory motion phantom

The Catalyst™ system (Fig. 1a), which was described by Hoisak et al. [28], includes three modules. In the test, the cRespiratory module was used for real-time motion tracking and gating radiotherapy. The sampling frequency of respiratory signals was more than 15 Hz. The appropriate scanning volume was selected in the Catalyst™ preset window, and camera parameters were adjusted to obtain images that met clinical requirements.

The QUASAR programmable respiratory motion phantom (Modus Medical Devices, London, ON, Canada) [22] was used to simulate respiratory curves. The phantom was modified in the experiment to explore the influence of amplitude variation on tracking accuracy (Fig. 1b). The phantom was placed vertically to move a translation stage along the anterior–posterior (AP) direction to simulate amplitude variation. In addition, a white plate was added as a detection plane, which was reinforced by two stents.

### Motion tracking accuracy of catalyst™

#### Test of camera thermal drifting

The three cameras of the Catalyst™ system were placed in boxes, and room temperature was stable. However, it is still necessary to verify the camera thermal drifting in the beginning. Once the cameras were powered on, the respiratory curves were recorded by the Catalyst™ system. The static thermal drift of the cameras and the stability of the real-time tracking was obtained through data analysis.

#### Tracking accuracy of cosine respiratory curves

The respiratory motion was simulated by the phantom according to following the formula [29]:

1 $$ Z(t)=-b\times {c\mathrm{o}s}^6\left(\pi t/\tau +\pi /2\right) $$

*b* and *τ* are the amplitude and period of a respiratory curve, respectively. To verify the impact of period and amplitude variations on the accuracy of real-time motion tracking, curves with periods ranging from 1 s to 10 s with an interval of 1 s and amplitudes ranging from 3 mm to 15 mm with an interval 2 mm were measured by the OSI system and then compared with the theoretical value calculated by the formula. Adjusted coefficient of determination (A_R^2^) and root mean square error (RMSE) were used to evaluate motion tracking accuracy. Owing to the modification of the phantom, motion accuracy was also evaluated by analyzing the differences between the input and output curves.

#### Tracking accuracy of respiratory curves of clinical patients

The actual respiratory signals of 13 patients were selected to test the motion tracking accuracy of the OSI system. These signals were divided into regular and irregular patterns and imported into the motion phantom. The motion curves recorded by the OSI system were compared to the respiratory curves output from the motion phantom. The subjective and manual overlapping of the curves causes a small error in spatial positioning, which leads to inaccurate real-time tracking. The Fourier transform [30] was applied to eliminate the influence of phase and spatial position on tracking accuracy [18]. Then, the curves were juxtaposed in the frequency domain and compared using the Pearson correlation coefficient (PCC) to quantitatively evaluate the similarity between the two data sets [18].

### Time delay of respiratory-gating radiotherapy

#### Test of time delay

**Fig. 2:**
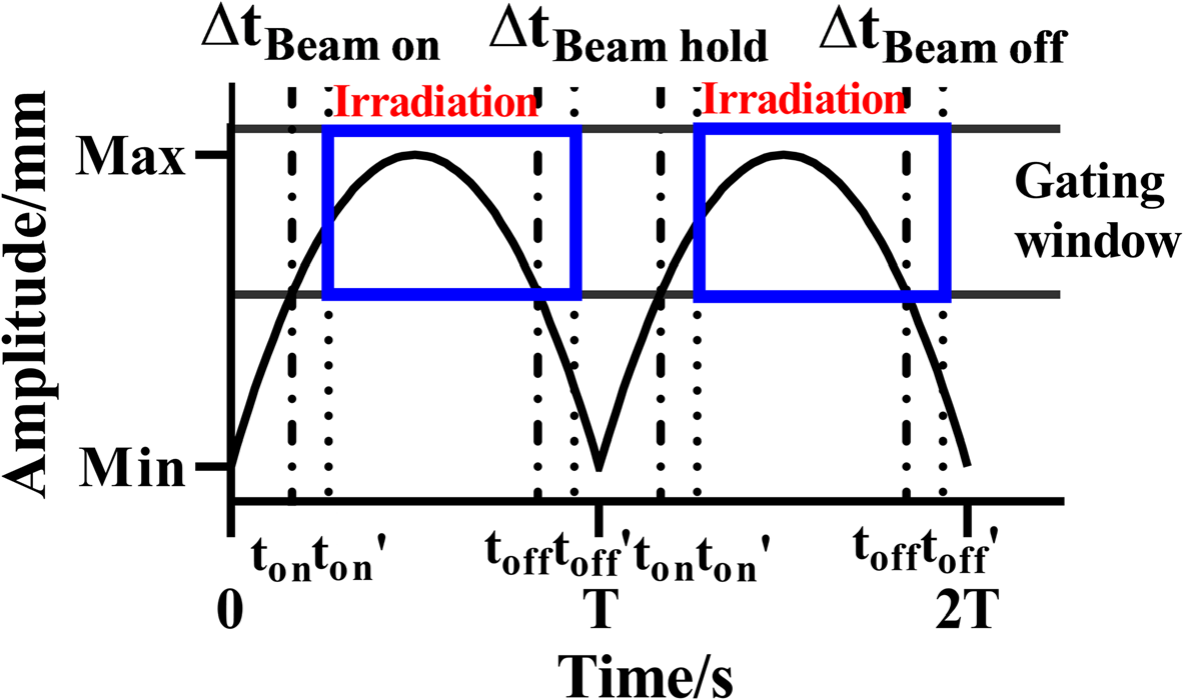
Measurement of time delay of amplitude-based respiratory gating. The gating signal is a uniformly accelerated curve. *t*_*on*_ and *t*_*off*_ represent the ideal case without time delay. *t*_*on*_*’* and *t*_*off*_*’* represent the actual case with system latency

Gating signals were simulated by the phantom and recorded by the Catalyst™ system. The gating level was set manually in a spatial gating window with a millimeter range. A gafchromic EBT3 (Ashland ISP Advanced Materials, NJ, USA) film was attached to the translation stage for dose measurement. The film was placed between two pieces of equivalent water material, each had a thickness of 2 cm, for dose build-up and providing backscatter. The phantom moved the film horizontally through a gated beam. Measurements were performed at the isocenter of a 2 × 2 cm^2^ field of 6 MV with 500 MU on the Versa-HD and Edge linacs. Figure 2 shows the measurement of the time delay. According to the accelerator characteristics, a period of 8 s and 6 s was tested on Versa-HD and Edge, respectively. The motion formula of the translation stage is

2 $$ y1=a\times {\left(t-T\right)}^2+b $$

*a* and *b* are coefficients, and *T* is half the movement period. The motion formula monitored by Catalyst™ is

3 $$ y2=\left(\mathit{\operatorname{Min}}-\mathit{\operatorname{Max}}\right)/{T}^2\times {\left(t-T\right)}^2+\mathit{\operatorname{Max}} $$

where *Min* and *Max* are the minimum and maximum values of the motion curve recorded by the Catalyst™ system, respectively.

To investigate the influence of the beam hold time on the time delay, the gating level was calculated according to eq. (3), with the beam hold time ranging from 409 ms to 4264 ms. For Elekta linacs, the Catalyst™ system software uses the minimum beam hold time of 3 s.

#### Film dosimetry

Film dosimetry was performed according to the self-developing procedure recommended by the film manufacturer. The EBT3 films were scanned in the 48 bit red-green-blue TIFF format using an Epson 11,000 XL scanner at 150 dpi in the professional mode with no image correction. As the post-coloration of the films can occur up to 6 h after irradiation [31], the films were scanned approximately 24 h after irradiation. Images were analyzed and converted into dose maps using the FilmQAPro® software (ISP Advanced Materials, New Jersey, USA).

#### Dose convolution-fitting method

**Fig. 3:**
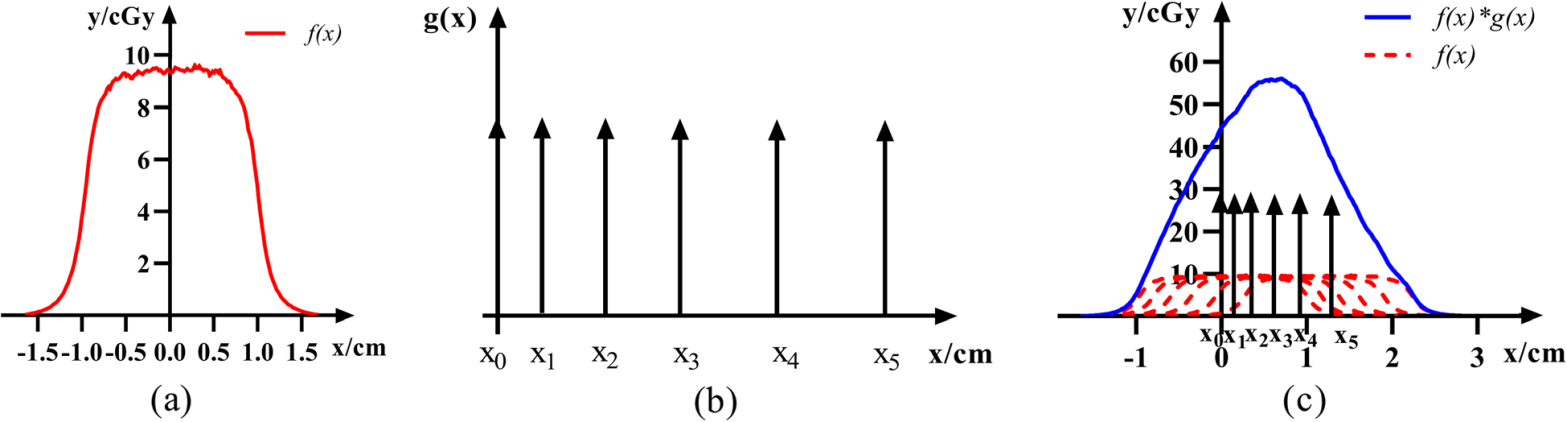
**a***f(x)* is the dose-profile fitting function for each pulse beam, and the position of the center axis of the field is the origin of coordinates. **b***g(x)* is comprised of Dirac delta function *δ*(*x*). The figure shows the graph of *g(x)* when *n* = 5 and *t*_*on*_ = 0. **c** Convolution of *f(x)* with *g(x)*, which reveals the basis of the dose-profile fitting by superimposing the dose at different points to obtain the final film dose profile

The dose convolution-fitting method simulates the pulse beam-on process of a linac, which simulates the actual formation process of the blackening of the film exposed to X-rays. The main principle of this method is shown in Fig. 3. Based on the dose profile of the film under the static state, the number of respiratory cycles and the time interval of the linac pulse signal (*∆t*) were combined to obtain the dose profile *f(x)* for each pulse beam (Fig. 3a). According to the motion formula of the film at different times,

4 $$ {x}_n=a\times {\left({t}_{on}+n\Delta t-T\right)}^2+b,n=0,1,2,3\cdots \left[\left({t}_{off}-{t}_{on}\right)/\Delta t\right] $$

*x*_*n*_ was combined with Dirac delta function *δ*(*x*) to generate the following new function (Fig. 3b):

5 $$ g(x)={\sum}_{n=0}^{\left[\left({t}_{off}-{t}_{on}\right)/\Delta  t\right]}\delta \left(x-{x}_n\right) $$

The total dose profile of the film was obtained by convolving functions *f(x)* and *g(x)* (Fig. 3c). Considering the times of beam on (*t*_*on*_*’*) and beam off (*t*_*off*_*’*) as variables, the appropriate variable interval was determined according to the theoretical time. A series of *t*_*on*_*’* and *t*_*off*_*’* values were obtained at a time interval of 10 ms. The least squares approach was used to evaluate the differences between the calculated and actual dose profiles of the film and find the optimal solution. The corresponding *t*_*on*_*’* and *t*_*off*_*’* were considered as the actual times of beam on and beam off. Then, the corresponding time delay was obtained through the theoretical time and calculated time.

## Results

### Camera thermal drifting

According to the fitted quadratic function trend line of the recorded data and its derivative curve, the thermal drift of the cameras stabilized after 17.2 min, with an average thermal drift of 0.12 mm (Fig. S1 Additional file 1). After stabilizing the thermal drifting of the cameras, the position recorded by the system remains stable, and no significant change is observed before and after the interruption. The fluctuation of the system record position is below 0.1 mm, which may be associated with the system noise.

### Tracking accuracy of cosine respiratory curves

**Fig. 4:**
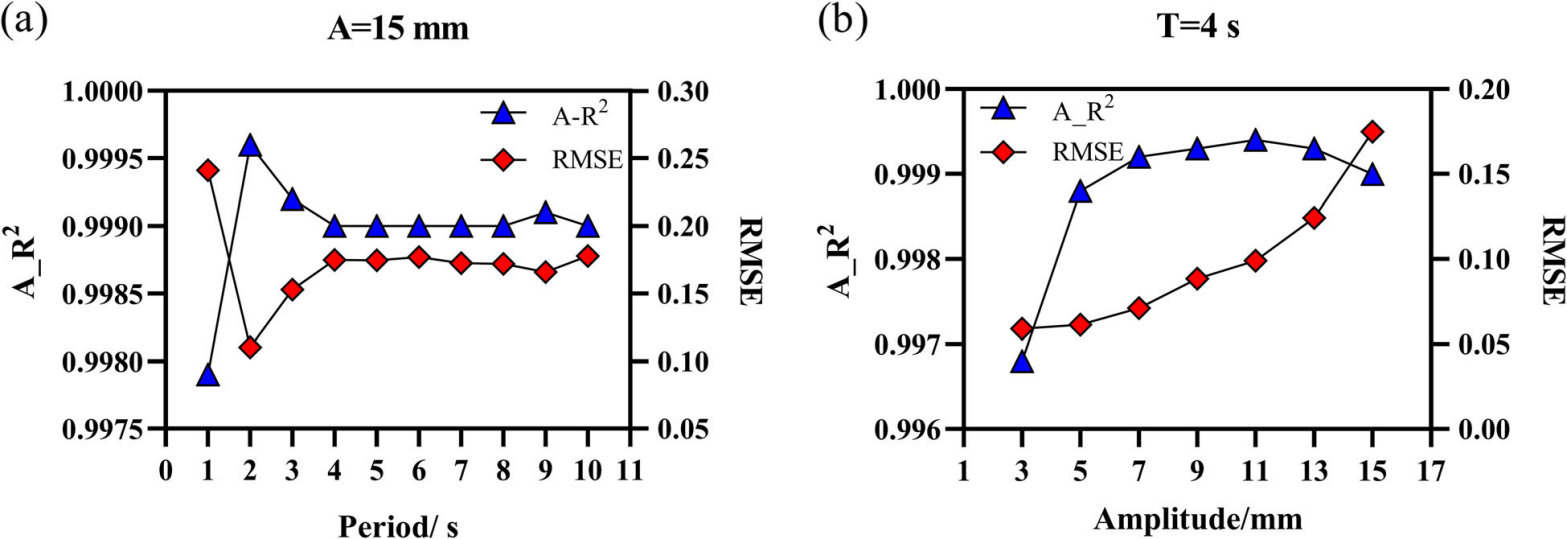
Value and trend of A_R^2^ and RMSE for cosine respiratory curves when (**a**) period and (**b**) amplitude change

The analysis of the input and output curves of the phantom showed that the maximum error was 0.03 mm. This indicates that the modified phantom does not affect motion accuracy. For all measured curves, A_R^2^ was larger than 0.996 and the RMSE was less than 0.25 (Fig. 4). This indicates that the system provides high real-time motion tracking accuracy and that period and amplitude variations have negligible influence on accuracy. The RMSE increased with amplitude and remained stable as the period increased. This shows that the system is more stable under period variations.

### Tracking accuracy of respiratory curves of clinical patients

The Fourier transform was applied to the respiratory curves recorded by the Catalyst™ system and phantom. The PCC of the curves in the frequency domain is shown in Fig. S2 (Additional file 2). The mean PCC for regular and irregular respiratory signals was 0.9985 (0.9971–0.9994) and 0.9983 (0.9955–0.9997), respectively, i.e., it was almost the same under different respiratory patterns. This indicates that the Catalyst™ system provides high real-time motion tracking accuracy for the respiratory curves of clinical patients.

**Fig. 5:**
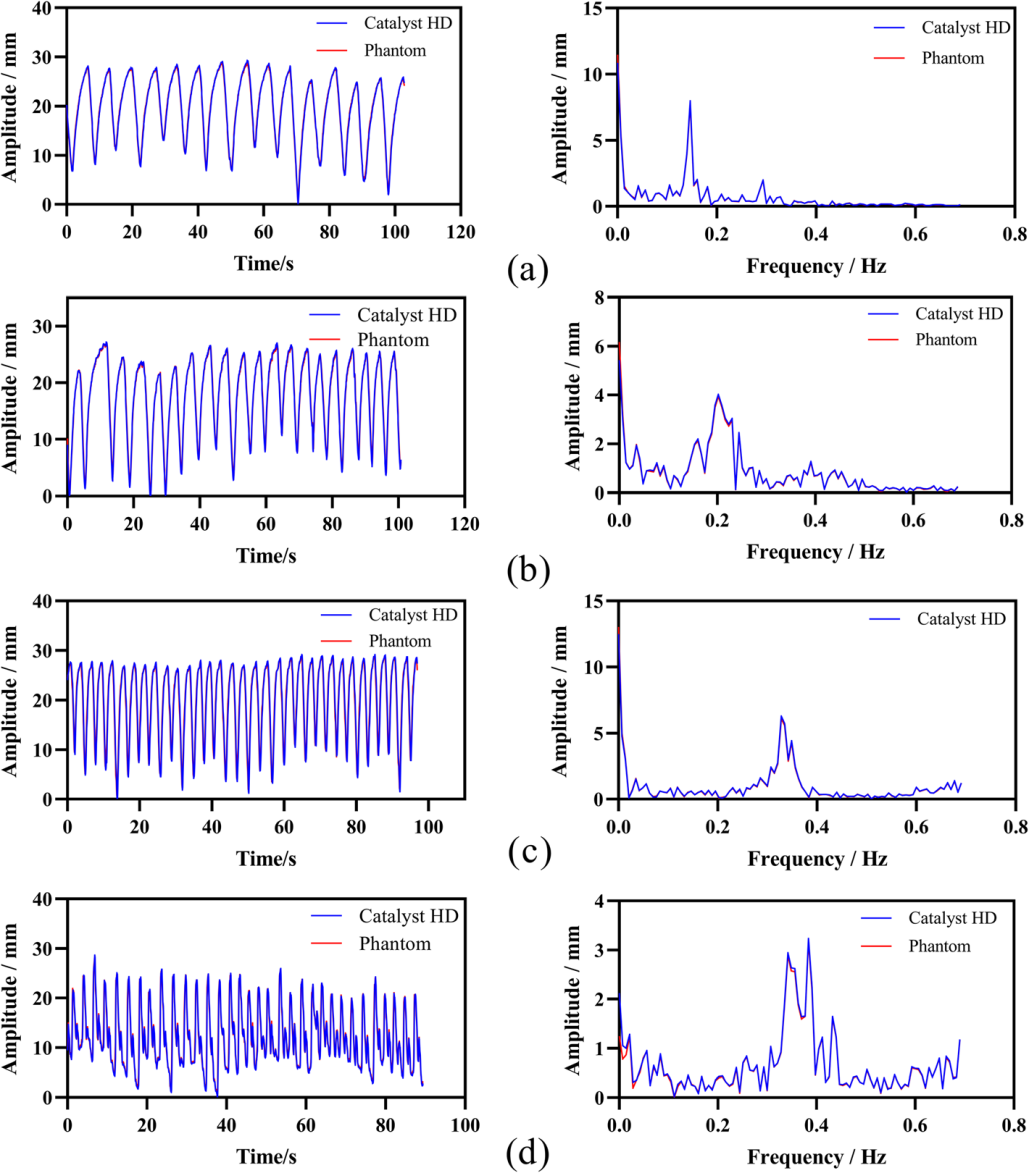
Four sample signals from the tested signals with spatial representation on the left and frequency representation on the right

The analysis showed that the frequency of clinical respiratory signals was mainly between 0 and 0.8 Hz (Fig. 5). The sampling frequency of the Catalyst™ system is approximately 15 Hz. According to the sampling theory, the system can reconstruct the actual respiratory signals of patients.

### Time delay of respiratory gating

**Fig. 6:**
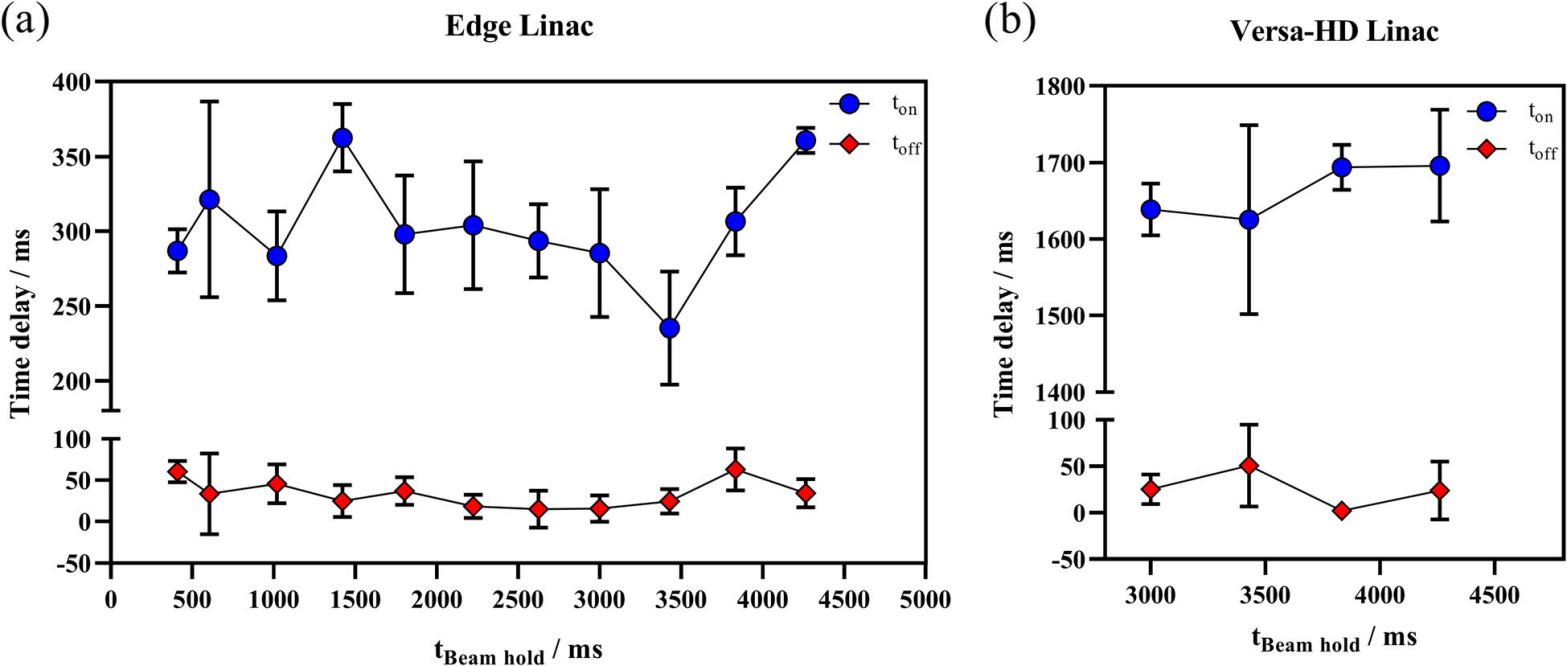
Time delay and its variation trend for (**a**) Edge and (**b**) Versa-HD with different beam hold times

In tested range of the beam hold time, the beam-on time delay was significantly larger than the beam-off time delay (Fig. 6). The beam-on time delay was approximately 303 ± 45 ms and 1664 ± 72 ms for Edge and Versa-HD, respectively. The corresponding beam-off time delay was approximately 34 ± 25 ms and 25 ± 30 ms for Edge and Versa-HD, respectively.

The fitting dose profiles exhibited good agreement with the actual film dose profiles (Fig. S3 Additional file 3).

## Discussion and conclusion

In this study, the respiratory motion tracking accuracy of Catalyst™ surface imaging system was investigated. The results demonstrate that the OSI system provides high real-time motion tracking accuracy with a PCC greater than 0.99. The average thermal drift of cameras was observed to be 0.12 mm in this study, which is similar to those reported by Shi et al. [19]. However, Stanley et al. [32] reported a higher thermal drift of 1 mm and a longer preheat time to reach stability. This may be due to the performance discrepancy of various cameras. Therefore, the thermal drift and preheat time of the OSI systems should be tested before clinical use. A possible reason for camera thermal drifting is that the imaging component of the Catalyst™ system is a charge coupled device whose signal receiving module works by converting an optical signal into a charge signal. In addition, the temperature of cameras changes the wavelength of signal light, which affects the amount of charge and eventually changes the position of detection.

At present, the method of film exposure is commonly used [25, 26, 33] to measure the gating time delay by combining the theoretical length of the blackening of the film with the actual length. Conventional film analysis methods mainly rely on the identification of the edges of the blackened parts of the film. Owing to the speed of film movement and the penumbra of the light beam, significant blurring of field edges can occur and the calculation of the time delay may be inaccurate. In this study, the calculation of the time delay by the dose convolution-fitting method does not depend on the recognition of edges, and the measurement accuracy of time delay can be effectively improved. However, this method obtains the average gating time delay during each measurement, so the fitting accuracy of the dose profile is affected by the stability of the respiratory gating time delay. Previous studies have shown that only a small variation exists at the start and end of gating time lags [21]. Our results have shown a good consistency between the fitted dose profiles and the measured dose profiles by films, indicating that this small variation in time delay does not affect the accuracy of the convolution fitting method.

Several studies on various gating devices have demonstrated that the time delays for different devices are different. The maximum differences in the beam-on and beam-off time delays are up to 270 ms and 485 ms, respectively [2, 21, 23, 24, 34]. In addition, for the same gating devices, the time delays obtained by different research centers are different [25, 35]. Our results indicate that for the same gating device, the time delay varies according to the linac. Therefore, the time delay for a new gating device needs to be measured on each linac before clinical use. In our test, the beam-on time delay of Versa-HD is considerably higher than that of Edge. The reason for this may be that an electron gun enters the standby mode instead of remaining active when Elekta linacs are in beam hold for a long time, and the gun requires considerable time to be in the stable activation mode again [35]. Moreover, for the Versa HD linac, the beam-on time delay could be minimized by changing certain parameters of the linac, such as the gun hold-on time [25]. In general, such adjustments can significantly reduce the beam-on time delay; however, it also reduces the electron gun lifetime [25].

The beam-on and beam-off time delays have different impacts on the curative effect of treatment. A large beam-on time delay significantly increases treatment time and reduces efficiency. For the same treatment plan, the treatment time of Versa-HD is much longer than that of Edge. In the case of a large beam-off time delay, normal tissues are exposed to high dose while the target does not receive adequate dose. The beam-off time delays of the linacs in this test are less than 100 ms. This meets the criteria of TG-142, i.e., there is no deviation between intended and delivered dose distributions and dose accuracy is not be influenced.

Our results show that the OSI technique can provide high accuracy for motion tracking in clinical applications despite the variations in the period and amplitude of respiratory signals. In addition, a dose convolution-fitting method has been proposed and validated, which can accurately measure the time delay of respiratory gating radiotherapy. The proposed method can be used to test the time delay of various respiratory gating devices. The evaluated OSI system for respiratory-gating radiotherapy offers a considerably longer the time delay of beam-on than beam-off. As the time delay for various OSI respiratory-gating systems may vary, it should be detected before clinical use.

## Supplementary information

**Additional file 1: Figure S1.** Trace plot for the first 30 min after the camera is plugged in and another 15 min after interruption by the rebooting of the Catalyst™ system. The y-axis shows the distance from the initial point.

**Additional file 2: Figure S2.** PCC calculated for the 13 respiratory signals with different respiratory patterns.

**Additional file 3: Figure S3.** Dose profiles calculated by dose convolution-fitting method and actual dose profiles under different beam hold times for (a) Versa-HD and (b) Edge.

## Data Availability

The datasets used during the current study are available from the corresponding author on reasonable request.

## Abbreviations

AP: Anterior–posterior
A_R^2^: Adjusted coefficient of determination
DIBH: Deep inspiration breath-hold
OSI: Optical surface imaging
PCC: Pearson correlation coefficient
RMSE: Root mean square error
SGRT: Surface-guided radiation therapy
