# Supplementary figures and images for: Accuracy of real-time respiratory motion tracking and time delay of gating radiotherapy based on optical surface imaging technique
Source: Radiat Oncol. 2020 Jul 10;15:170. doi: 10.1186/s13014-020-01611-6 (PMC7350729; doi:10.1186/s13014-020-01611-6)

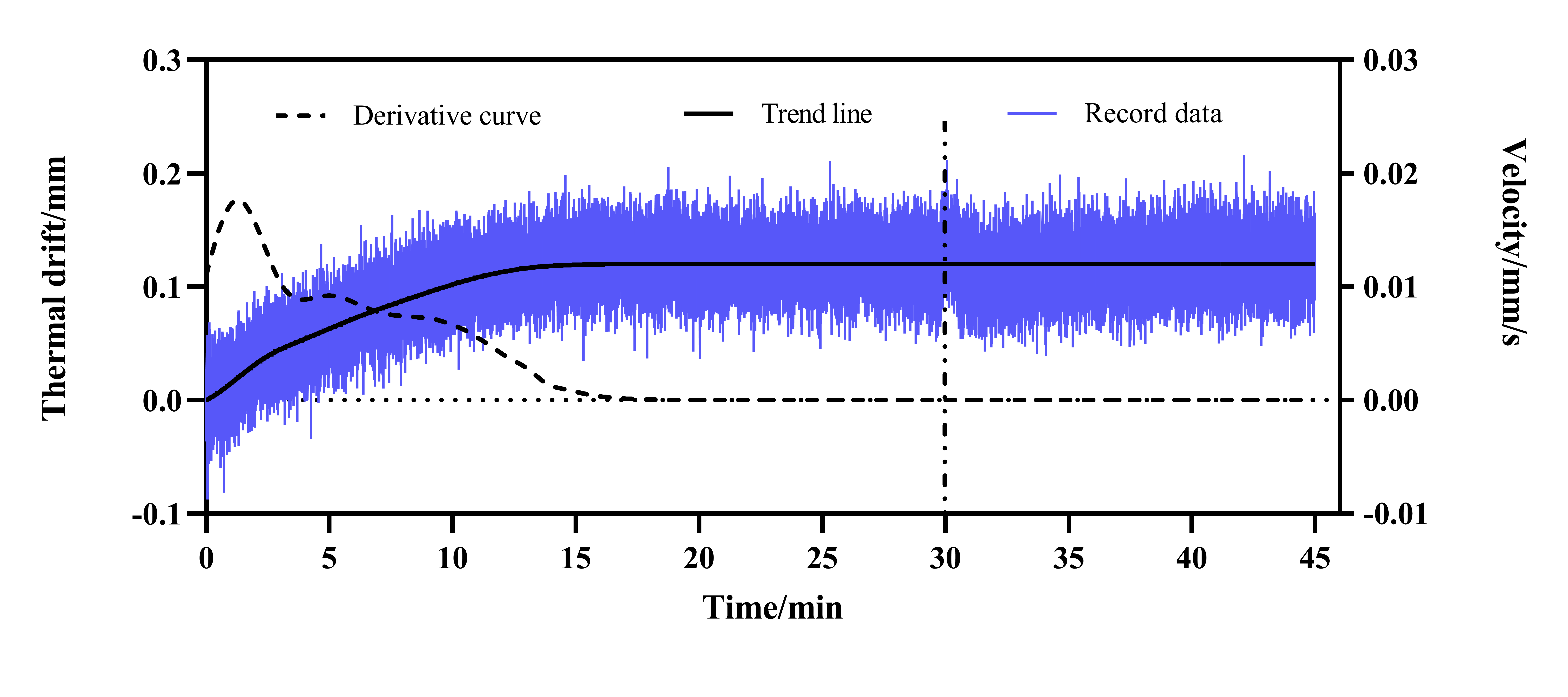

Supplement: Supplementary file 1 — Additional file 1: Figure S1. Trace plot for the first 30 min after the camera is plugged in and another 15 min after interruption by the rebooting of the Catalyst™ system. The y-axis shows the distance from the initial point. [file 13014_2020_1611_MOESM1_ESM.tif]

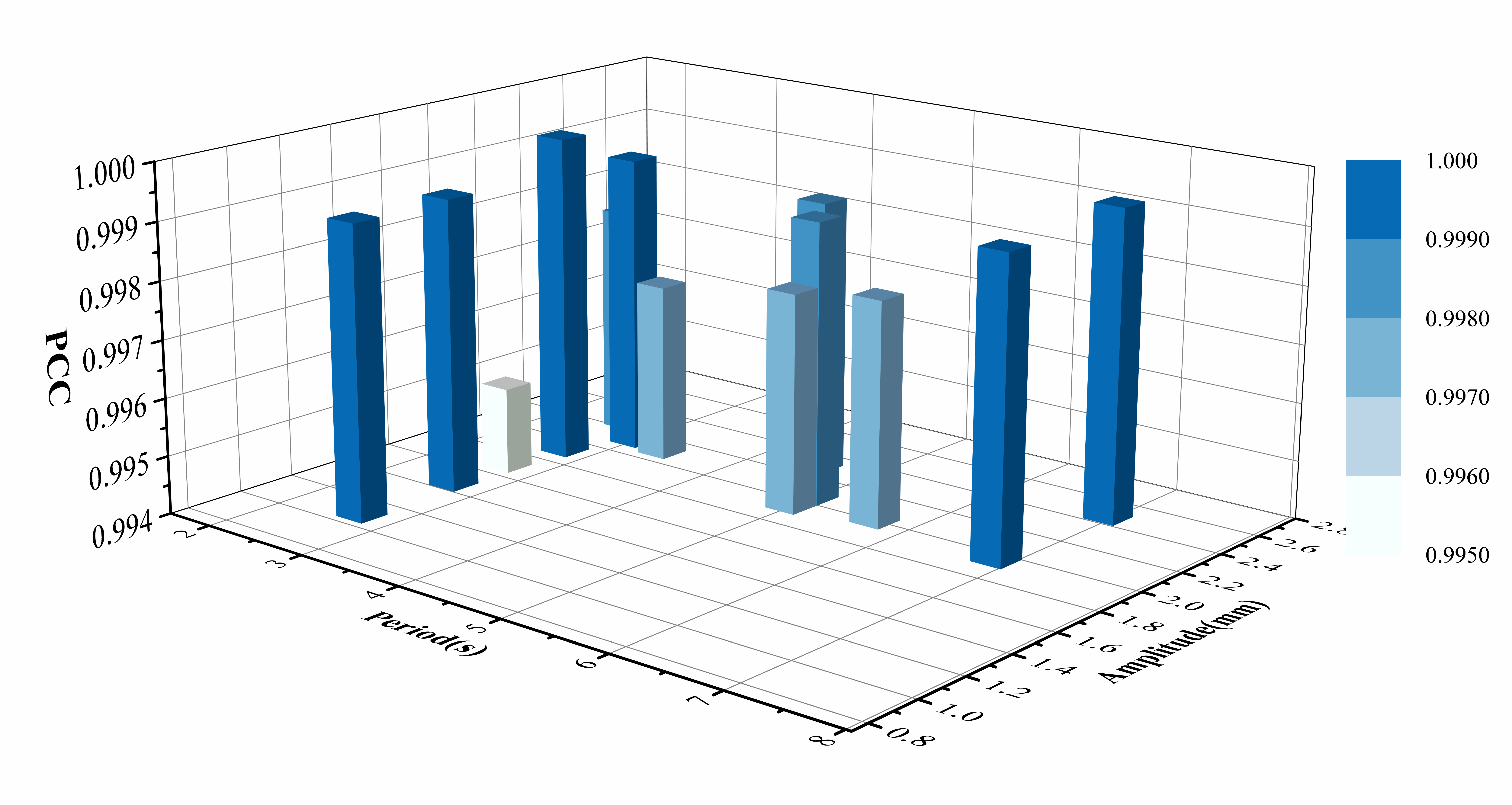

Supplement: Supplementary file 2 — Additional file 2: Figure S2. PCC calculated for the 13 respiratory signals with different respiratory patterns. [file 13014_2020_1611_MOESM2_ESM.tiff]

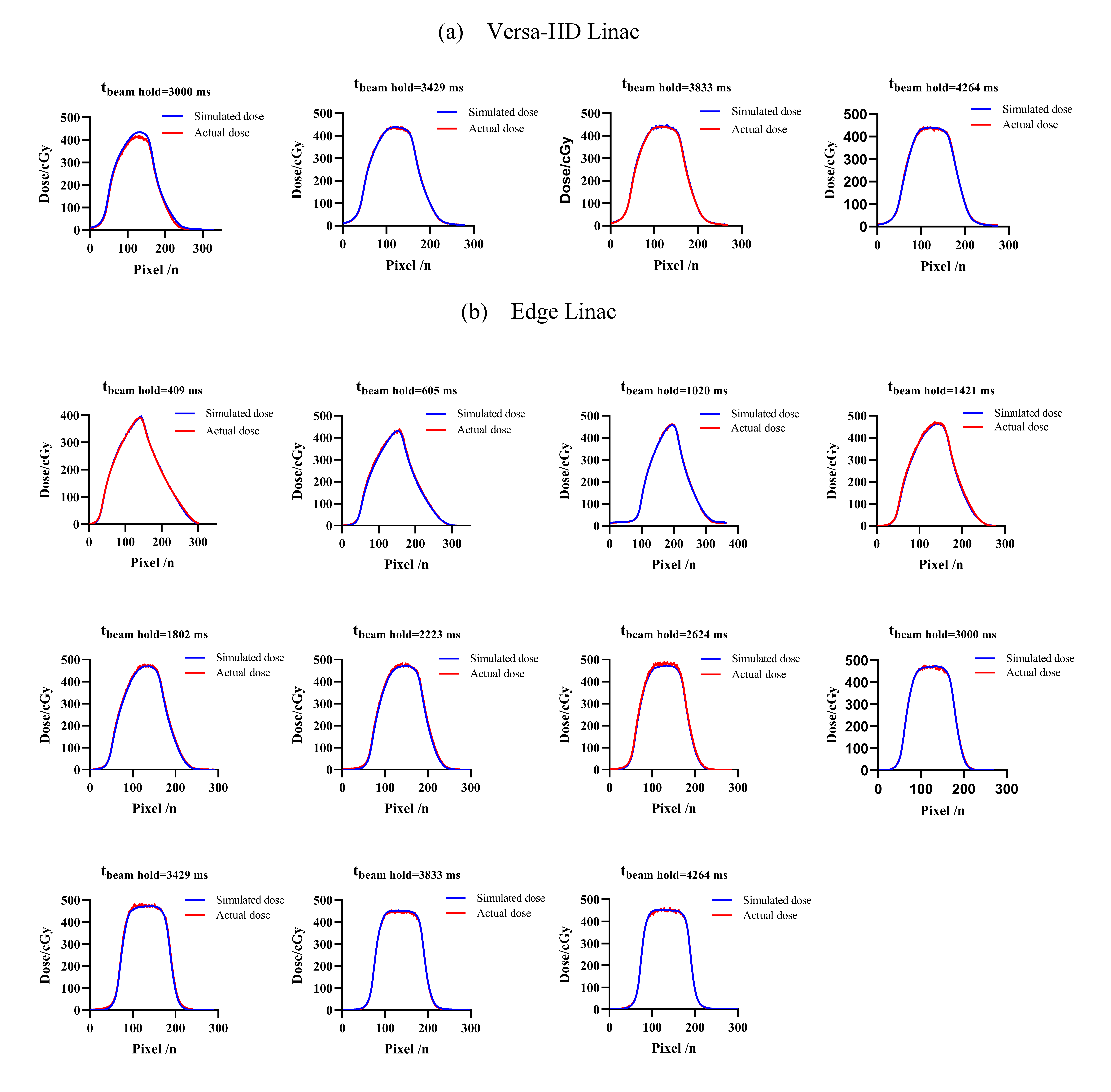

Supplement: Supplementary file 3 — Additional file 3: Figure S3. Dose profiles calculated by dose convolution-fitting method and actual dose profiles under different beam hold times for (a) Versa-HD and (b) Edge. [file 13014_2020_1611_MOESM3_ESM.tif]
